# Development and evaluation of machine learning algorithms for the prediction of opioid-related deaths among UK patients with non-cancer pain

**DOI:** 10.1371/journal.pdig.0001190

**Published:** 2026-01-27

**Authors:** Jose Benitez-Aurioles, Carlos Raul Ramirez Medina, David Jenkins, Niels Peek, Meghna Jani

**Affiliations:** 1 Division of Informatics, Imaging and Data Science, University of Manchester, Manchester, United Kingdom; 2 Centre for Epidemiology Versus Arthritis, Centre for Musculoskeletal Research, University of Manchester, Manchester, United Kingdom; 3 The Healthcare Improvement Studies Institute, Department of Public Health and Primary Care, University of Cambridge, Cambridge, United Kingdom; 4 Salford Royal Hospital, Northern Care Alliance, United Kingdom; 5 NIHR Manchester Biomedical Research Centre, Manchester University NHS Foundation Trust, Manchester Academic Health Science Centre, United KingdomManchester, United Kingdom; Liverpool John Moores University - City Campus: Liverpool John Moores University, UNITED KINGDOM OF GREAT BRITAIN AND NORTHERN IRELAND

## Abstract

The global rise in prescription opioid use has contributed to an opioid epidemic, associated harms, and unintentional deaths in several western countries. Opioids however continue to be regularly prescribed for acute pain and in the chronic pain context due to limited treatment options. Currently there are no accurate tools that help predict which patients prescribed opioids may be at risk of death, which depends on the cultural context and varies across countries. Existing models do not account for statistical considerations such as censoring and competing risks. Using nationally representative data from the United Kingdom from 1,026,139 patients newly prescribed an opioid, we developed three competing risk time-to-event models: a regression model, a random forest, and a deep neural network to predict opioid-related deaths using UK primary care records. The models were externally validated in an external cohort of 337,015 patients. The models exhibited good discrimination and positive predictive value during internal validation (C-statistic for the regression model, random forest, and neural network: 84.3%, 84.4% and 82.1% respectively), and external validation (C-statistic for the regression model, random forest, and neural network: 81.8%, 81.5% and 81.5% respectively). Prior substance abuse, lung and liver comorbidities, morphine, fentanyl, or oxycodone at initiation and co-prescription of gabapentinoids were some of candidate predictors associated with a higher risk of opioid-related mortality within the models. These results demonstrate how routinely collected data from a nationally representative dataset may be used to develop and validate opioids risk algorithms to better help clinicians and patients predict risk to this serious adverse outcome.

## Author summary

The rising use of prescription opioids has led to serious health concerns, including a growing number of preventable deaths. Yet, opioids remain a common treatment for pain, leaving doctors and patients with a difficult balance between benefits and risks. There are no accurate tools to identify which patients prescribed opioids for non-cancer pain may face a higher risk of dying from them. Using electronic health records from over one million people in the United Kingdom who were newly prescribed an opioid, we created and tested three different prediction models, including different types of machine learning. We were able to accurately define opioid-associated deaths from information presented on death certificates. These models used information such as medical history, other medications taken at the same time, and the type/ dose of opioid prescribed to estimate an individual’s risk of opioid-related death. We found that all three models performed well, both when tested on the original data and when tested on a separate group of patients. We developed and validated risk prediction tools to predict the most serious adverse event to opioids for the first time using nationally representative data. These could help guide safer prescribing decisions and support conversations between patients and healthcare professionals about opioid use.

## Introduction

For the past 20 years, there have been concerns of a considerable increase in the prescription of opioids for non-cancer related pain in countries such as US Canada and the UK [1–6]. Accompanying this surge, the clinical awareness of opioid-related adverse effects has increased, in line with the increase in prescribing. The concurrent growth of opioid consumption, both legal and illegal, and opioid-related harms has been termed the opioids epidemic and has been a leading cause of death for young adults in the US. Reducing opioid prescribing for non-cancer pain to reduce premature mortality and other associated harms is a National Health Service (NHS) England medicines optimisation priority for 2024/25 [7]. Despite national efforts to prevent harm, the UK continues to experience very high rates of drug related deaths, with recent evidence suggesting that UK mortality is worse than other high-income countries [8]. Age standardised mortality rate for deaths in the UK related to drug poisoning has risen every year since 2012, with just under half of all drug-related deaths registered in 2024 confirmed to involve an opioid [9].

Previous studies have identified factors associated with opioid-related adverse events including the type of opioids prescribed, concurrent medication, demographics, and several mental disorders on a population level [10–13]. Given limited treatment options for pain management, opioids continue to be prescribed for acute and chronic pain. However, it is not currently possible to predict which patients may develop the most serious adverse outcomes. Being able to do so could provide prescribers the ability to tailor more effective care by determining the need for heightened monitoring, the co-prescription of risk-mitigating medication such as naloxone, re-evaluation of treatment plans or prioritisation of effective biopsychosocial interventions if the risk is too high to prescribe such drugs at all. There has been increasing interest in developing prognostic clinical prediction models for outcomes for opioid associated harms, yet nearly all have been in North America using administrative data and none have yet been widely implemented [14,15].

Supervised machine learning (ML) methods leverage large amounts of data to extract complex relationships between variables [16]. ML could be used for predicting adverse outcomes for individual patients by harnessing the large databases of patient information being generated in healthcare systems, such as in electronic health records (EHRs). In the past decade, the application of ML in clinical risk prediction has expanded, in some cases achieving better performance than regression models in areas such as emergency admission or readmission risk [17–19]. Additionally, ML methods could potentially better estimate the risk of rarer outcomes by better modelling the risk profile of individuals with multiple risk factors. On the other hand, whether ML models outperform regression models for clinical prognosis in general has been put into question, with potentially biased performance estimates found in the reporting of many ML models [20].

Of particular interest are the new developments of time-to-event ML models [21]. When predicting long-term outcomes, not censoring time when a patient leaves the study, e.g., due to being lost to follow-up, can introduce biases into a model’s predictions [22]. Time-to-event models, such as a Cox regression models allows the estimation of the probability of the event over time. Such models are important considerations in opioids prognostic research, as unlike traditional classification models that predict a binary outcome (e.g., event/no event), time-to-event models estimate the risk of an event occurring at different time points. In addition, competing risk models [23] consider the potential misestimation of the primary outcome due to the censorship of competing risk events, such as deaths unrelated to opioids. If a patient dies from another cause (a competing risk), they can no longer experience opioid-associated death. Therefore, ignoring competing risks may inflate the estimated risk because the model treats individuals who die from other causes as if they were still at risk of opioid-related death. Compared to regression models, competing risk time-to-event ML models provide the possibility to study non-linear and complex relationships between variables, associating patterns in a patient’s data with non-proportional survival curves [24–27]. A recent systematic literature review on machine learning applications in predicting opioid-associated adverse events to date reported that time-to event or competing risk models were not performed and an area for future development [15].

While there is existing research on the application of ML in predicting adverse opioid outcomes, the use of ML time-to-event analysis for this task has not yet been explored. Additionally, previous ML opioids outcome work has mostly been carried out in US patient data, where the cultural context of opioid prescribing and opioid-related adverse events are different from other countries, such as the UK.

Our study aims to evaluate the potential of statistical modelling and ML algorithms in predicting opioid-related deaths among patients initiating opioid use in the UK, and to determine whether regression or ML models perform better when predicting mortality risk due to opioids. The results of this study could help prescribers identify those at the highest risk of opioid-related deaths and allow patients to make better informed decisions based on their individual risk.

## Methods

### Data source

In this retrospective cohort study, we used data from the Clinical Practice Research Datalink (CPRD) Gold (between 1^st^ of January 2006 and 31^st^ of Dec 2017) and Aurum (between January 1st, 2015, to October 31st, 2021) for model training and validation respectively. CPRD is a longitudinal database of anonymized primary care electronic health records (EHRs) from over 14 million patients registered with a GP in the UK [28,29]. CPRD GOLD contains data contributed by practices using Vision software, whilst CPRD AURUM contains data from the EMIS Web electronic patient record system software. The demographic composition of the CPRD population is representative of the UK population with regards to age, sex, and ethnicity. CPRD contains both diagnostic and electronic prescribing information for each patient, recorded through Read codes and prescribing codes. A Read Code is a standardised clinical coding system used in UK primary care to represent diagnoses and symptoms, while a prescription code is a standardised identifier used to classify and record prescribed medications within EHRs. CPRD also allows individual linkage to death records of the Office of National Statistics (ONS). Cause of death information was specifically requested and approved for this study, which captures this information from the patient’s death certificate. Additionally, the data were linked to the Townsend Deprivation Index, a score of the deprivation index derived from the patient’s postcode [30].

### Study population and design

**Fig 1:**
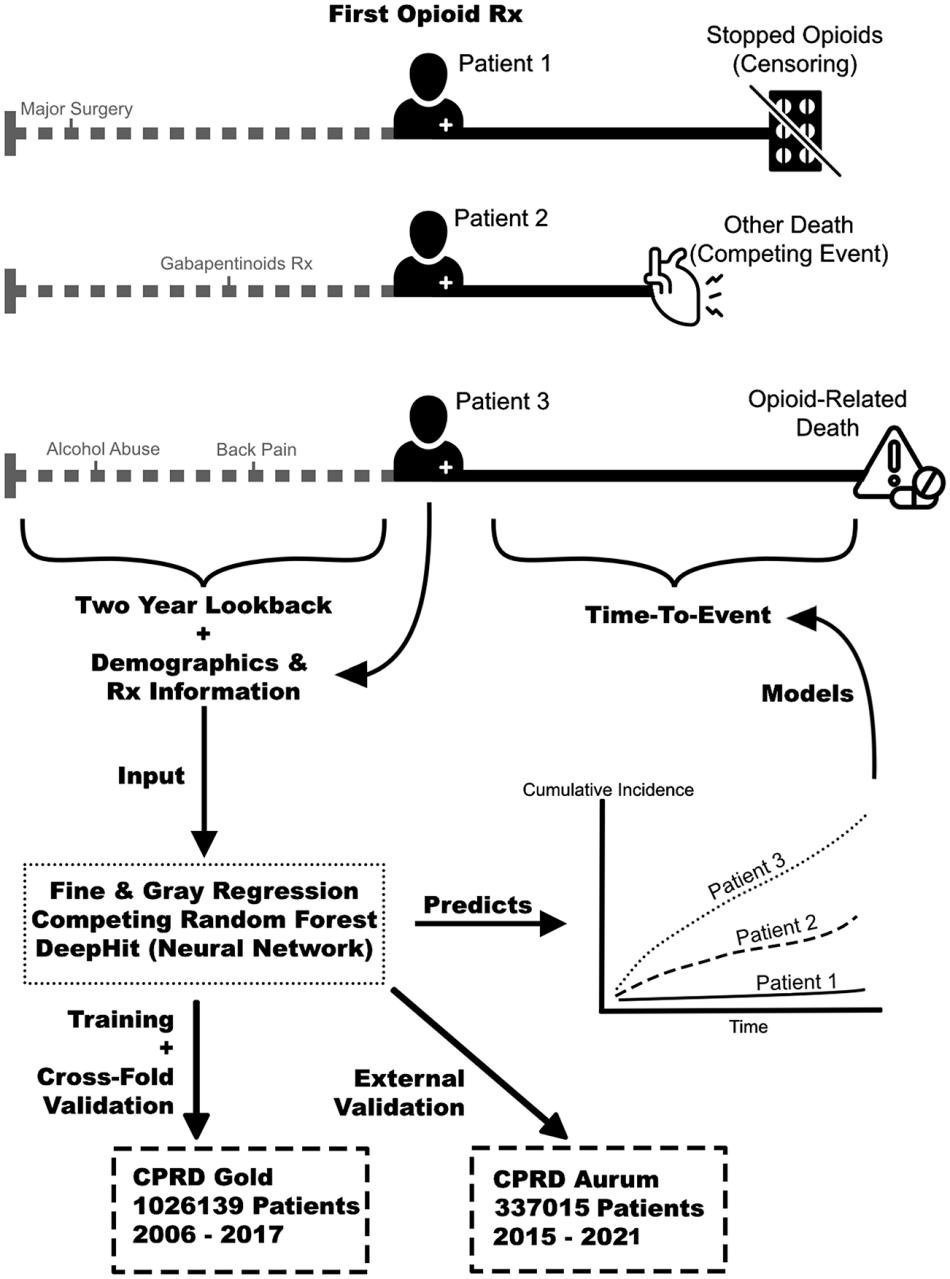
Overview diagram of the study design. The cohort includes patients at opioid initiation. The candidate predictors are assessed at the time of the first prescription (type of opioid, demographics), and by using a two-year lookback period on their primary care records. The regression and machine learning models are trained to predict the cumulative incidence functions of the time-to-event outcomes, and are validated internally and in a second, external dataset.

Adults over the age of 18 who were new users of opioids without cancer from CPRD Gold were included. New patients taking opioids were defined as patients who had not been prescribed opioids in at least two years preceding the incident (or first) opioid prescription. The index date was defined as the date the first opioid was prescribed. Individuals who had a history of cancer within the previous ten years prior to opioid initiation, were not included in the analysis, due to distinct opioid prescribing mechanisms and a different baseline risk of death. To do this we excluded patients with a Read codes for any malignancy with the exception of those with non-melanoma skin cancer prior to their index opioid prescription, as previously described [3,5]. Additionally, patients who were prescribed an opioid within six months of a cancer diagnosis were excluded to reduce the risk of protopathic bias, when an opioid may be prescribed inadvertently for an early manifestation of cancer before it has been detected diagnostically (e.g., pain or cough preceding a diagnosis of cancer). Patients who were prescribed methadone or oral buprenorphine either two years before or at the index date were also excluded, as patients in the UK are often prescribed these drugs to treat opioid use disorder secondary to recreational/ illicit use, for which the baseline risk markedly different than that of the general population. Buprenorphine patches however are frequently used in the U.K. for pain management and therefore included. We did not rely on opioid use disorder diagnosis codes for exclusion, as it is inconsistently coded in CPRD and medication records offer a more reliable indicator of active treatment. CPRD measures electronic prescribing data, and all other opioid prescriptions were considered in the analysis, to make the analysis and population of interest as inclusive as possible. Patients with data considered not to be up-to-standard according to the quality checks performed by CPRD were excluded. The study design is shown in Fig 1. The CPRD drug exposure data were transparently prepared using a previously published ‘drug preparation algorithm’ [31], with decisions made for this work outlined in S1 Text.

Patients were censored at their death, if transferred out of the practice, two years after the index date, or when the practice ceased meeting CPRD data-quality standards. ONS linkage was used to identify both the patient death date and cause of death. Opioid-induced deaths were identified using ICD-10 codes and classified as such if any of these codes appeared as either the underlying cause or a contributory cause on the death certificate. Opioid associated deaths are underrepresented using ONS data as coroners’ reports (if conducted at all) are not available through any means for such primary care data. Restricting classification to the underlying cause alone or using a narrower ICD-10 definition would substantially reduce the number of outcomes and limit statistical feasibility. Deaths not attributable to opioids were treated as competing events. Full codelists are provided in S1 Table.

To reduce the likelihood of overfitting to the data, we performed a sample size estimation for a time-to-event model as developed by Riley *et al* [32], using as performance baseline the survival model trained by Glanz *et al* [33] (S1 Text). This calculation is intended to give the maximum number of parameters that a regression model can use without risking overfitting the model to the training data. For our study, the maximum number of parameters recommended to be used was 216, more than three times the candidate parameters considered by our models.

Predictor variables were chosen based on prior scientific literature and clinical relevance. They included patient demographics, comorbidities (included within the Charlson comorbidity score), other types of prescriptions such as benzodiazepines and gabapentinoids, healthcare utilisation history, type of opioid being prescribed at initiation and morphine milligram equivalents (MME) per day at initiation (S1 Text). MME/day was calculated using cumulative daily dose of opioid multiplied by potency of the opioid according to per the conversion ratios specified by the U.S. Centre for Disease Control and Prevention [34]. Diagnoses were extracted from Read codes during a baseline assessment period and additional medications from Product codes, two years prior to the incident opioid prescription. Missing values (Ethnicity and Townsend score in CPRD Gold, and Ethnicity and Region of the Practice in CPRD Aurum) were handled using multiple imputation with chained equations (MICE) [35], using the MICE package in R [36]. Ethnicity and deprivation scores are known to be variables with a higher proportion of missingness in CPRD, therefore multiple imputation was performed to reduce potential bias associated with a complete case analysis. Imputation was performed within each cross-validation fold. For each fold, a single imputed dataset was generated with 50 iterations per chain. To account for the possibility that missingness itself was informative, we additionally created a missingness indicator for each variable with missing data and included it as a predictor alongside the imputed value to model informative missingness for the prediction [37]. This allowed the models to retain all individuals while simultaneously capturing any predictive signal associated with missingness.

### Statistical analysis

We trained three models of varying complexity to account for competing risks in time-to-event prediction:

1. The Fine & Gray model [38] adapts Cox regression to account for competing risk by building hazard sub-distributions. We introduce LASSO penalization in order to prevent model overfitting.
2. The Competing Random Forest model [27] uses nonparametric random survival forests to estimate the cumulative incidence functions of each event type. Number of trees, per-tree sample size and variable size, number of random splits and minimum size of terminal node were controlled to prevent overfitting.
3. The DeepHit model [24] builds a neural network to estimate the cumulative incidence functions of each event type. It is composed of a shared and a cause specific network to estimate the joint distribution of all competing events. Learning rate, total epochs trained, width and length of shared and individual networks, class imbalance sampling rate and dropout rate were controlled to prevent overfitting.

Further details on model hyperparameter tuning and model training can be found in S1 Text. The Fine & Gray model was implemented in R using the packages survival [39] and glmnet [40]. The Random Forest model was implemented with R and the package randomForestSRC [41]. The DeepHit model was implemented with Python and the libraries PyTorch [42], PyTorch Lightning [43] and PyCox [44].

The three models were validated using 5-fold cross-validation, as it provides more accurate estimates than split sampling [45]. As the DeepHit model oversamples positive outcomes to improve its performance, it is expected to be initially mis-calibrated, and thus all three model are recalibrated to be well-calibrated to their training data, the inner fold in the cross-validation, before being validated on the outer fold. The performance was evaluated at three different prediction horizons: 6, 12, and 24 months after index data (i.e., when the patient was prescribed their first opioid). For each round of the cross-validation, the model only had access to the inner fold until the validation step, and since only first recorded incidences were included, no patient was in both the inner and outer fold.

Discriminative performance was estimated using the standard receiving operator characteristic (ROC) curve along with the precision-recall (PR) curve. The area under the ROC curve (AUROC) and area under the PR curve (AUPRC) were also calculated and plotted for all prediction horizons between 3 and 24 months.

Calibration performance was evaluated using quintile calibration plots, weighted with pseudo-observations to account for the other mortality competing risk [46]. The average cumulative incidence function of each model is compared to that observed in the population, reporting the average expected to observed risk ratio of the model. In addition, the calibration slope was calculated for all prediction horizons by fitting a linear model to the five quintiles calibration data of the calibration plots.

Furthermore, for the three prediction horizons of interest, patients were stratified based on their risk level. Patients were classified as being moderate risk (above a cut-off risk chosen by the Youden index) or high risk (in the 5th percentile of prediction scores). The recall, specificity, positive predictive value, number of patients needed to screen a positive case (defined as the inverse of the positive predictive value) was also calculated in both groups for the regression model and for the higher performing ML model (according to the AUROC and AUPRC).

Final models were trained and recalibrated to the entirety of the data. The coefficients of the Fine & Gray regression were converted into hazards ratios. Furthermore, Shapley Additive exPlanations (SHAP) values [47] for patients who had experienced opioid-related death of the higher performing ML model were calculated. SHAP values use game theory principles to explain the output of a predictive model by attributing importance scores to each input variable used in the model, thus indicating how much each feature contributed to the final prediction. The use of SHAP values is particularly relevant for “black box” models, where the decision-making process may be opaque or difficult to interpret. For each individual, the SHAP value for a particular variable implies that for this patient’s value, the risk of experiencing an opioid-related death is increased or reduced by the SHAP value, as compared to what their risk would be had they had the overall average of that variable. Although in different scales, a qualitative comparison of the order of both the Fine & Gray hazard ratios and the SHAP values of the DeepHit model (for predicting the risk 24 months after the first opioids prescription) was carried out to better understand the differences in decision-making of the two models.

The use of the models is highlighted through example cases of a random patient in the bottom half, one in the top half, one in the top ten percent, and one in the top one percent of risk predictions for the DeepHit model. The predictions of the three models are given, as well as the top predictive variables for the patient as given by the SHAP values of the DeepHit model.

The checklists for the transparent reporting of a multivariable prediction model for individual prognosis or diagnosis (TRIPOD) [48] and the checklist for the minimum information about clinical artificial intelligence modelling (MI-CLAIM) [49], are included in S2 Table Equator checklists. Detailed guidance on the interpretation of clinical risk prediction models and terminology is provided in provided elsewhere [48,50].

### Ethics

The study was approved by the CPRD’s Independent Scientific Advisory Committee (approval number: 23_002658).

## Results

### Baseline characteristics

Data from 1,026,139 and 337,015 patients who contributed to 2,350,730 and 781,362 patient-years of follow up were included in the development and validation cohorts. The median follow-up for both cohorts was 730 days, and the corresponding frequency of the outcome were 0.12% (corresponding 1,226 individuals) and 0.09% (293) respectively. Competing deaths constituted 5.1% (52,665) of deaths in the development cohort and 5.9% (19,849). Baseline characteristics of the patients can be found in Table 1.

**Table 1:** Characteristics of the cohort used for developing (CPRD Gold) and validating (CPRD Aurum) the prediction models.

Characteristics	CPRD Gold (n = 1,026,139)	CPRD Aurum (External Validation data) (n = 337,015)
Female n (%)	591,149 (57.6%)	191,464 (56.8%)
Median Age (IQR^†^)	50 (36 - 66)	58 (45 - 70)
Ethnicity, n (%)		
Asian	42,368 (4.1%)	18,482 (5.5%)
Black	24,317 (2.4%)	12,380 (3.7%)
Mixed	7,726 (0.8%)	2,558 (0.8%)
White	867,304 (84.5%)	24,1851 (71.8%)
Other	15,159 (1.5%)	6,078 (1.8%)
Missing	69,265 (6.8%)	55,666 (16.5%)
Region n (%)		
East Midlands	26,470 (2.6%)	7,637 (2.3%)
East of England	105,020 (10.2%)	14,154 (4.2%)
London	129,803 (12.6%)	60,207 (17.9%)
North East	24,045 (2.3%)	14,757 (4.4%)
North West	168,288 (16.4%)	64,535 (19.1%)
South Central	131,936 (12.9%)	0 (0%)
South East Coast	141,647 (13.8%)	54,743 (16.2%)
South West	139,363 (13.6%)	41,992 (12.5%)
West Midlands	122,721 (12.0%)	59,646 (17.7%)
Yorkshire and the Humber	36,846 (3.6%)	15,085 (4.5%)
Missing	0 (0%)	4,259 (1.3%)
Townsend Quintile n (%)		
1^st^ (least deprived)	213,694 (20.8%)	65,015 (19.3%)
2nd	216,253 (21.1%)	66,580 (19.8%)
3rd	215,018 (21.0%)	65,635 (19.5%)
4th	219,107 (21.4%)	70,330 (20.9%)
5th	161,253 (15.7%)	69,165 (20.5%)
Missing	814 (0.1%)	290 (0.1%)
Smoking Status n (%)		
Never Smoked	125,542 (12.2%)	148,986 (44.2%)
Current Smoker	149,064 (14.5%)	97,349 (28.9%)
Former Smoker	751,533 (73.2%)	90,680 (26.9%)
Charlson Score n (%)		
0	722,018 (70.4%)	224,797 (66.7%)
1	199,745 (19.5%)	76,939 (22.8%)
> 1	104,376 (10.2%)	35,279 (10.5%)
Major Surgery n (%)	28,859 (2.8%)	18,658 (5.5%)
Alcohol Abuse n (%)	22,233 (2.2%)	5,238 (1.6%)
Substance Use Disorder n. (%)	15,401 (1.5%)	1,624 (0.5%)
Depression n (%)	231,636 (22.6%)	26,437 (7.8%)
Attempted Suicide/Self-Harm history n (%)	34,741 (3.4%)	4,044 (1.2%)
Median Number of GP Visits in Last Year (IQR^†^)	7 (4 - 11)	7 (4 - 11)
Median Number of Healthcare Utilisation in Last Year (IQR^†^)	10 (6 - 17)	19 (10 - 32)
Opioid at Initiation n (%)		
Buprenorphine patches	5,621 (0.5%)	1,522 (0.5%)
Codeine	737,860 (71.9%)	289,340 (85.9%)
Dextropropoxyphene	2,140 (0.2%)	24 (0.0%)
Diamorphine	2,670 (0.3%)	589 (0.2%)
Dihydrocodeine	172,690 (16.8%)	30,433 (9.0%)
Fentanyl	3,155 (0.3%)	336 (0.1%)
Morphine	11,911 (1.2%)	4,781 (1.4%)
Oxycodone	2,127 (0.2%)	645 (0.2%)
Tramadol	92,737 (9.0%)	8,966 (2.7%)
Other	2,332 (0.2%)	379 (0.1%)

Percentages are rounded and might not always sum to 100%. † Interquartile range. Abbreviations; IQR, interquartile range; n, number

The patients in both cohorts were more likely to be female (58% and 57% for CPRD Gold and Aurum respectively) and white (85% and 72%), being representative of all UK regions (although no South-Central England practice were available for CPRD Aurum) and socioeconomic deprivation levels. The median age for incident users was 50 and 58 years for CPRD Gold and Aurum, respectively. Codeine (72% and 86%), dihydrocodeine (17% and 9%) and tramadol (9% and 3%) were the most common initial opioid types prescribed.

In terms of differences, the validation cohort was more ethnically diverse (15% vs 28% of non white or unknown ethnicity), had higher smoking rates (15% for CPRD Gold and 29% for CPRD Aurum), lower depression rates (23% and 8%) and experienced a higher number of median healthcare utilisations (10 and 19).

In the development dataset, missing data was found in two categorical variables: the ethnicity variable, with 6.8% missing, and the Townsend score, with <0.1% missing, and were replaced with a missing marker. There was other no missing data in the continuous values within the patients included (age, number of GP visits and health utilisations). In the validation data, missingness was found in the ethnicity variable, with 16.5% missing, the region of the patient’s GP practice, with 1.3% missing, and the quintile of the Townsend score, with <0.1% missing. Missing data was present in two continuous values: the total number of GP visits in the last year with 5.4% missing, and the total number of health utilisations with <0.1% missing (due to incomplete records). Ethnicity and Townsend score in CPRD Gold, and ethnicity and region of the practice in CPRD Aurum were imputed as described in the methods.

### Model development

The development of the Fine & Gray model, the Random Forest, and the DeepHit model, including the five-fold cross-validation, optimisation, and training of the final model, took 120, 140, and 90 hours, respectively. The final Fine & Gray model had 34 non-zero parameters. The Random Forest had a total of 241 trees, with at most 5 variables split per node and a minimum node size of 724. The DeepHit network had a total of 48,500 parameters, with two shared layers for all risks and a single individual layer for each three outcomes (opioid-related death, competing death, and other censoring), all with a width of 32 nodes.

### Discrimination performance

**Fig 2:**
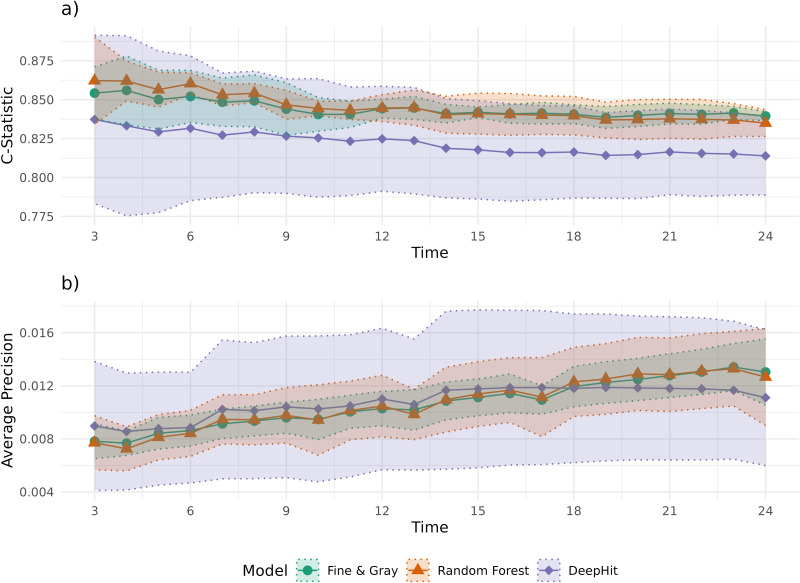
Average C-Statistic for the Fine & Gray model, random forest, and DeepHit across the first 24 months of prediction horizon (a). Average precision (positive predictive value) across different true positive rates for the Fine & Gray model, random forest, and DeepHit across the first 24 months of prediction horizon **(b)**. Confidence intervals are shown as dotted lines.

Discriminative performance, as seen through the C-Statistic over time (Fig 2a) was comparable between The Fine & Gray model (Average C-statistic over prediction horizons between months 3–24 of follow-up: 84.3%, 95% CI: 83.6%-85.1%) and the Random Forest model (84.4%, 95% CI: 83.6%-85.3%). The DeepHit exhibited slightly lower discriminative performance of the three models (82.1%, 95% CI: 78.7%-85.6%). In terms of the average AUPRC (Fig 2b), the Fine & Gray model (1.1%, 95%CI: 0.9%-1.2%), Random Forest (1.1%, 95%CI: 0.9%-1.3%) and DeepHit (Average AUPRC between months 3–24 of follow-up: 1.1%, 95%CI: 0.6%-1.6%) had similar performance. This was above the baseline value for the AUPRC (i.e., the average precision over the same timeframe of a model that is no better than chance) of 0.1%.

### Calibration performance

**Fig 3:**
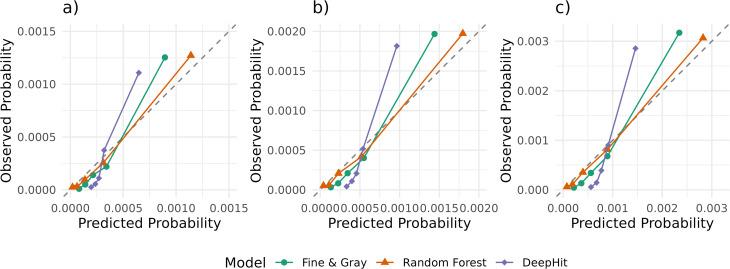
Calibration plots for the Fine & Gray model, random forest, and DeepHit when predicting 6-month risk of opioid-related death (a). Calibration plots for the Fine & Gray model, random forest, and DeepHit when predicting 12-month risk of opioid-related death **(b)**. Calibration plots for the Fine & Gray model, random forest, and DeepHit when predicting 24-month risk of opioid-related death **(c)**.

In terms of the average expected to observed risk ratio, the Fine & Gray model (Average Ratio between 3 and 24 months of follow-up: 1.00, 95%CI: 0.91 - 1.09) and the Random Forest (1.00, 95%CI: 0.91 - 1.09) were well-calibrated. After recalibration with the training data, DeepHit (1.00, 95%CI: 0.91 - 1.09) was also well-calibrated. Calibration plots over different prediction horizons are shown in Fig 3. At 6 months, the calibration slopes of the Fine & Gray model, random forest, and DeepHit model were 0.85 (95%CI: 0.77-0.94), 1.24 (95%CI: 1.02-1.46) and 1.42 (95%CI: 0.72-2.13) respectively. At 12 months, they are 0.86 (95%CI: 0.76-0.95), 1.30 (95%CI: 1.03-1.57) and 1.56 (95%CI: 0.78-2.34), and at 24 months, 0.82 (95%CI: 0.75-0.90), 1.22 (95%CI: 1.04-1.41) and 1.26 (95%CI: 0.83-1.69).

### Risk stratifications

Table 2 shows the stratification of the patient population for different risk thresholds for the Fine & Gray and Random Forest models. For both thresholds, the performance of the two models was comparable, and neither performed significantly better than the other.

**Table 2:** Performance metrics of the Fine & Gray regression, competing random forest and Deep Hit at the classifying threshold chosen by identifying the Youden index and by identifying the 5^th^ percentile of risk scores for 6-months, 12-months and 24-months prediction horizons. 95% confidence intervals are presented in brackets.

Youden	6 Months			12 Months			24 Months		
	Fine & Grey	Random Forest	Deep Hit	Fine & Grey	Random Forest	Deep Hit	Fine & Grey	Random Forest	Deep Hit
Recall (%)	77.5 (72.3 - 82.6)	79.3 (75.3 - 83.2)	73.9 (71.5 - 76.4)	76.5 (71.4 - 81.5)	77.3 (74.7 - 79.9)	79.8 (77.1 - 82.4)	75.1 (70.7 - 79.4)	78.2 (75.7 - 80.7)	77.0 (73.5 - 80.6)
Specificity (%)	74.1 (69.9 - 78.4)	71.0 (66.9 - 75.0)	74.1 (67.9 - 80.3)	76.2 (72.1 - 80.3)	74.0 (72.4 - 75.7)	70.6 (65.9 - 75.3)	78.1 (74.7 - 81.5)	74.9 (73.0 - 76.9)	73.5 (69.7 - 77.3)
Positive Predictive Value (%)	0.39 (0.35 - 0.43)	0.36 (0.31 - 0.40)	0.39 (0.30 - 0.47)	0.46 (0.41 - 0.51)	0.42 (0.40 - 0.43)	0.39 (0.32 - 0.46)	0.56 (0.50 - 0.62)	0.50 (0.46 - 0.55)	0.48 (0.41 - 0.54)
Number Needed to Screen^†^	258 (230 - 285)	284 (249 - 319)	271 (209 - 334)	222 (198 - 246)	241 (230 - 252)	266 (217 - 314)	181 (160 - 203)	200 (181 - 219)	215 (184 - 246)
Patients Flagged as Positive (%)	26.2 (21.9 - 30.5)	29.3 (25.3 - 33.4)	26.2 (20.1 - 32.4)	24.4 (20.2 - 28.6)	26.5 (24.8 - 28.2)	30.1 (25.5 - 34.7)	23.2 (19.5 - 26.9)	26.3 (24.3 - 28.3)	28.0 (24.2 - 31.8)
High-Risk	**6 Months**			**12 Months**			**24 Months**		
	**Fine & Grey**	**Random Forest**	**Deep Hit**	**Fine & Grey**	**Random Forest**	**Deep Hit**	**Fine & Grey**	**Random Forest**	**Deep Hit**
Recall (%)	34.1 (31.2 - 37.0)	26.6 (17.1 - 36.0)	26.6 (17.1 - 36.0)	36.0 (34.1 - 38.0)	29.0 (18.9 - 39.0)	29.0 (18.9 - 39.0)	36.0 (32.9 - 39.1)	28.3 (19.3 - 37.3)	28.3 (19.3 - 37.3)
Specificity (%)	95.1 (95.1 - 95.1)	95.1 (95.0 - 95.1)	95.1 (95.0 - 95.1)	95.2 (95.2 - 95.2)	95.1 (95.0 - 95.2)	95.1 (95.0 - 95.2)	95.4 (95.2 - 95.5)	95.2 (95.0 - 95.5)	95.2 (95.0 - 95.5)
Positive Predictive Value (%)	0.89 (0.81 - 0.97)	0.70 (0.44 - 0.95)	0.70 (0.44 - 0.95)	1.03 (0.97 - 1.09)	0.83 (0.53 - 1.13)	0.83 (0.53 - 1.13)	1.24 (1.14 - 1.33)	0.97 (0.64 - 1.30)	0.97 (0.64 - 1.30)
Number Needed to Screen^†^	113 (103 - 123)	204 (42 - 366)	204 (42 - 366)	97 (91 - 103)	168 (40 - 296)	168 (40 - 296)	81 (75 - 88)	134 (45 - 223)	134 (45 - 223)
Patients Flagged as positive (%)	5	5	5	5	5	5	5	5	5

High risk is defined as in the 5th percentile of prediction scores, ^†^Defined as the average number of individuals needed to be screened by the model in order to detect one positive case. It is equivalent to the inverse of the positive predictive value.

### Model interpretation

**Fig 4:**
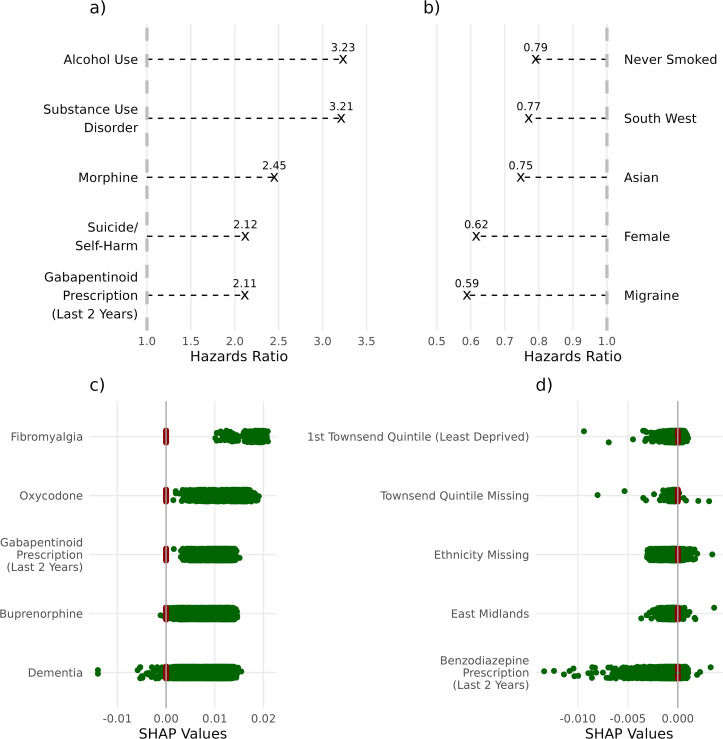
Hazard ratio boxplot of the 5 largest (a) and 5 smallest (b) coefficients of the Fine & Gray regression (a). SHAP plots of the DeepHit model, with the 5 largest (c) and 5 smallest (d) average differences between positive (green) and negative (red) individuals plotted. A full list of the Fine & Gray coefficients and full list of the SHAP differences of the DeepHit model are available in the S3 Tables. Suicide/self-harm refers to attempted suicide or self-harm history.

For the Fine & Gray model coefficients (Fig 4a, complete list in S3 Table 1 in S3 Table), the 5 coefficients associated with the largest increase in the prediction of the model for categorical variables were alcohol abuse (Hazards Ratio: 3.23), substance use disorder (3.21), receiving a morphine prescription at initiation (2.45), attempted suicide/self-harm (2.12) and having a gabapentinoids prescription in the last two years (2.11). The coefficients associated with the largest decreases were never having smoked (0.79), living in the Southwest of England (0.77), being Asian (0.75), being female (0.62) and a history of migraines (0.59).

For the SHAP values of the DeepHit neural network (Fig 4b, complete list in S3 Table 2 in S3 Table, and SHAP plot for continuous values in S3 Fig 1 in S3 Table), the five categorical variables with the highest risk SHAP values (as determined by the difference between the average of the SHAP value of individuals with and without the positive value of the categorical variable) were fibromyalgia (SHAP Difference: 0.018), receiving a oxycodone prescription at initiation (0.012), having a gabapentinoids prescription in the last two years (0.010), receiving a buprenorphine prescription at initiation (0.008) and dementia (0.008). The 5 variables with the lowest risk SHAP values were being part of a GP practice in Yorkshire and the Humber (-0.00024), having ethnicity missing from your records (-0.00043), having a missing Townsend score (-0.00047), being part of a GP practice in the East Midlands (-0.00050) and having a benzodiazepines prescription in the last two years (-0.051). Diamorphine (0.005), fentanyl (0.004) and morphine (0.003) prescriptions at initiation were in the top ten of features with the highest associated SHAP difference.

### Clinical examples

We have included the predictions of 4 patients across the risk score spectrum, alongside the predicted values of the three models, and the highlighted top predictors according to SHAP DeepHit values, in S3 Table 3 in S3 Table.

### External validation

**Fig 5:**
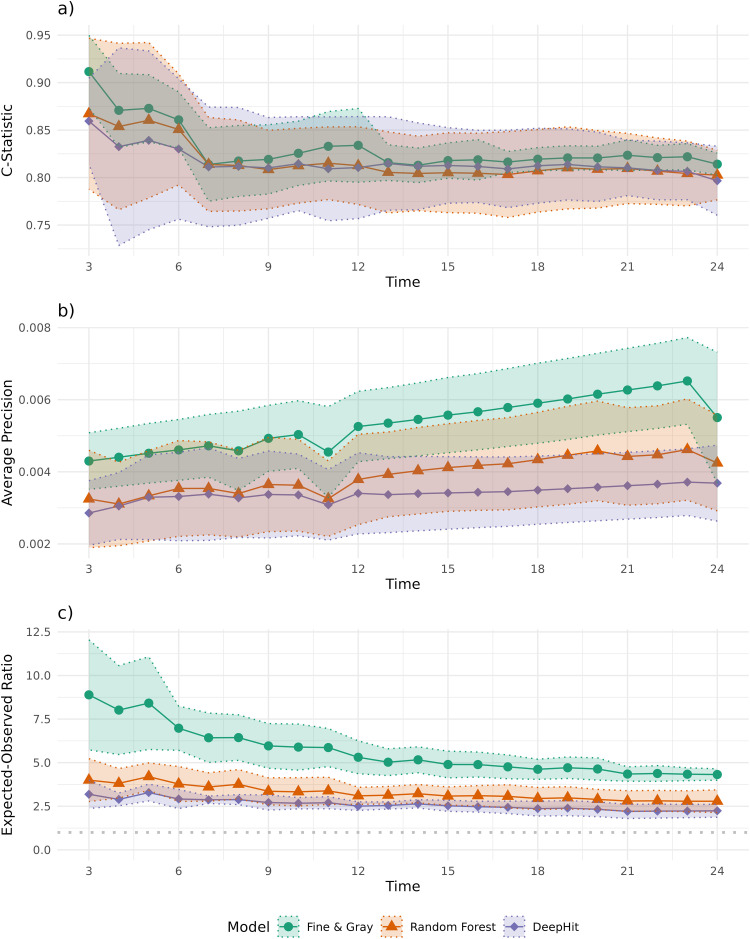
Performance measures of the three models in the external validation dataset. Average C-Statistic for the Fine & Gray model, random forest, and DeepHit across months 3 to 24 of prediction horizon **(a)**. Average precision (positive predictive value) across different true positive rates for the Fine & Gray model, random forest, and DeepHit across months 3 to 24 of prediction horizon **(b)**. Expected/Observed ratio for the Fine & Gray regression, random forest, and DeepHit across the months 3 to 24 of prediction horizon, with a grey dotted line shing the ideal expected/observed ratio of 1 **(c)**. Confidence intervals are shown as dotted lines.

The Fine & Gray, random forest, and DeepHit models had an average AUROC (Fig 5a) over months 3–24 of 81.76 (95%CI Using 500 Bootstraps: 75.90-87.62), 81.49 (95%CI: 77.60-85.38) and 81.37 (95%CI: 76.49-86.26) respectively, and an average AUPRC (Fig 5b) of 0.52% (95%CI: 0.39%-0.66%), 0.39% (95%CI: 0.27%-0.52%) and 0.34% (95%CI: 0.24%-0.44%), where the baseline AUPRC is 0.04%. According to both metrics the Fine & Gray model shows superior discrimination performance in the new dataset.

In terms of calibration, the average expected to observed ratio at 6 months was 7.57 (95%CI: 5.67-9.48), 3.78 (95%CI: 2.78-4.78), and 2.92 (95%CI: 2.37-3.46) for the Fine & Gray, Random Forest and DeepHit models, respectively (Fig 5c). At 12 months, it was 5.71 (95%CI: 4.62-6.80), 3.10 (95%CI: 2.61-3.59), and 2.51 (95%CI: 2.26-2.75), and at 24 months, it was 4.49 (95%CI: 3.99-5.00), 2.79 (95%CI: 2.14-3.44), and 2.24 (95%CI: 1.87-2.61). At 6 months, the calibration slope was 0.32 (95%CI: 0.28-0.35), 1.06 (95%CI: 0.77-1.35), and 1.13 (95%CI: 0.88-1.38), at 12, 0.32 (95%CI: 0.29-0.36), 0.93 (95%CI: 0.72-1.14), and 0.94 (95%CI: 0.70-1.19), and at 24, 0.33 (95%CI: 0.30-0.35), 0.90 (95%CI: 0.77-1.02), 0.57 (95%CI: 0.51-0.63). The random forest and DeepHit models were better calibrated in the new dataset compared to the Fine & Gray model, but still showed overall poor calibration. Additional performance metrics are presented in S3 Tables 4–6 in S3 Table.

## Discussion

We developed a series of time-to-event clinical prediction models employing both regression and machine learning methodologies to predict opioid-related deaths among a cohort of patients initiating opioids. We built three competing risk prediction models of varying complexity, training and internally validating these on separate UK cohorts. In the internal validation, the machine learning models had comparable performance to the Fine & Gray regression model. In the external validation, the Fine & Gray model showed the best performance in terms of discriminative issues, illustrating the stability of regression methods.

### Strengths and limitations

This is the first study assessing the potential of ML in relation to opioid-related mortality in the United Kingdom, developing, and externally validating these models using large, nationally representative cohorts using real-world primary care records. This supports the generalisability of the models and results to the entirety of the United Kingdom. This study is amongst one of the few which have explored the potential of opioid risk prediction outside of North America [15]. In addition, harnesses variables that are widely available in primary care EHRs rather than from prospective observational studies, facilitating the potential implementation of these models in clinical practice. Through ONS linkage, this study ascertained cause-specific mortality related to opioids as mentioned on the death certificate, rather than all-cause mortality, strengthening this analysis.

There are a limited number of studies comparing regression and ML models in a competing risk time-to-event framework [15,51]. This is the first study applying survival ML models to predict adverse opioid outcomes. By using interpretability techniques such as SHAP values, we were able to explore the prediction mechanisms used by the neural network. Overall, the variables in DeepHit considered to be important were the same as those used by Fine & Gray, with substance use abuse, alcohol disorder, morphine prescriptions and high levels of deprivation being associated with increases of risk in the decisions of the models. These features are consistent with previous work done in smaller retrospective studies in the US [52]. We also performed an external validation on a separate group of patients in CPRD Aurum. However, differences between the training and external validation data can have consequences for model performance even though performed in the same setting due to temporal changes in prescribing and coding practices.

The study needs to be interpreted in the context of its limitations. Large and representative, EHRs, such as CPRD Gold and Aurum, may reflect some inherent limitations related to the mechanisms of routine data recording [53,54]. As a result, the evaluation of clinical utility using decision curve analysis was inherently limited. In low-prevalence settings, decision curves tend to lie close to or overlap the “treat none” strategy across most of the threshold ranges, as differences in net benefit require much larger samples to estimate with acceptable precision. To address this limitation, we additionally reported complementary measures of clinical utility, such as sensitivity, specificity, PPV and number needed to screen at different prediction horizons, as recommended by reporting checklists for clinical AI models such as MI-CLAIM [49]. Due to the limited sample size and rarity of opioid-related deaths, more detailed temporal validation was also not statistically feasible. Access to linked UK addiction centre data was not available as part of this data source.

While CPRD measures electronic prescribing information, dispensing or administration information was not available. We therefore used a previously published drug preparation algorithm [31] to transparently prepare the such data and communicate the decisions made during this process (S1 Text). Codeine is the most frequently prescribed opioid the dataset, reflecting UK primary care prescribing practices compared to other countries [3,5]. Previous work has shown that the most common indications for opioids in the UK are musculoskeletal disorders such as osteoarthritis and low back pain, while respiratory indications such as cough are less common [55]. In addition, although the MME/day at initiation was included as a candidate predictor, the evolution of the dose after initiation was not considered. This was to prevent data leakage, when the model has access to information that would not be available at the time of prediction. Total MME/day at the time of the event has been associated with mortality in opioid-treated patients, with MME/day of <50 per day associated with lower risk, compared to higher doses [56]. This study focuses on prediction at the time of opioid initiation, however the use of dynamic predictions using prescription history and cumulative dose as predictor could be explored in further work.

It is likely that opioid-related deaths in the UK are underreported in ONS records as the primary cause of death, reflecting under recognition of such events in clinical practice. In the UK post-mortems are conducted by the coroner for specific indications such as following a sudden, violent or unexpected deaths [57] and reports are not linked with CPRD to protect the patient’s identity. For this reason, we used a broader definition of what constitutes as an opioid-related death, considering both underlying and contributory causes. However, even after applying this broader definition, the outcome of interest remained infrequent.

In this study, we focussed on the validation of three models of increasing complexity. We, however, did not explore other emerging methodologies such as transformer-based [58] or long short-term memory networks [59], as such models do not yet have the same level of maturity for time to event prediction with as the three models that we used. We considered that the DeepHit model had enough complexity to leverage potentially useful high-level relationships between predictors, but further work could explore alternative models for opioid-related death risk prediction in the presence of competing events. In addition, we were restricted our inclusion of predictors as outlined by our sample size calculation and therefore prioritised a knowledge driven rather than data-driven approach in choosing candidate predictors. The advantage of doing so includes reducing the likelihood of overfitting to a specific dataset and developing a clinically interpretable model that is less likely to be influenced by spurious associations. The final model uses a hybrid approach where the clinically relevant variables are retained in the final model following data-driven refinement. However, we acknowledge that a limitation is that the novel variables that could improve model performance further, using a data driven approach may have been overlooked.

The interpretation of coefficients and SHAP values of the models of this work is limited, as this study was designed as a predictive investigation rather than an aetiological one, precluding the drawing of any causal conclusion about the data. It is important to ensure that the associated features in the model are not interpreted as causal, as would be the case in any risk prediction model. We included variables that were correlated and while their inclusion enhances predictive performance it does not allow causal interpretation of the calculated associations. However, the investigation of coefficients still can be valuable, to better understand the decision-making of models and address the ‘black box’ of ML models [60]. Finally, differences in missingness patterns between GOLD and AURUM may influence changes in model performance and calibration, and the impact of different imputation techniques should be explored in future work.

### Comparisons to other studies and practice implications

Researchers have developed prediction models to predict a diverse range of opioid-related adverse outcomes, leveraging administrative claims data [61], EHRs [33], prescription monitoring [62], mainly sourced from North America [15]. Moreover, ML models for opioid-related adverse outcome prediction, with models such as random forests [63,64], boosting trees [63,14] and neural network architectures [63,65,66] have generally demonstrated superior performance compared to logistic regression. These have, so far, all been binary classification problems, where censoring and competing risks were not considered, something which is potentially important in the prediction of opioid-related outcomes.

In this study, well-defined structured variables were used as the input of all models, with the aim of evaluating whether more complex ML models could uncover complicated relationships between these variables to improve their predictive power. ML and deep learning techniques have sometimes been utilized to instead extract information directly from unstructured data sources, such ‘unprocessed’ EHRs. These methods can leverage clinical free text using natural language processing techniques [67,68], or sequences of clinical codes as recorded in the system [65,69]. Although promising, the use of unstructured clinical data for risk assessment is particularly sensitive to generalisability issue, as models can more easily leverage patterns that are only specific to a hospital, system, or a particular moment in time [70]. Practically, such unstructured data are not widely accessible through curated data sources such as CPRD, due to information governance regulations and the risk of patient reidentification through free text.

During internal validation, the models accurately estimated the overall risk of opioid-related death in the population, but predicted probabilities were too close to the overall average risk. In the external validation cohort, the models overestimated the risk of opioid-related death on average. This calibration deficiency is in part due to the rarity of the event of interest and is a consequence of evaluating cause-specific mortality, instead of all-cause mortality. The external calibration issues could also potentially be attributed to the distributional shifts particularly salient in EHRs, caused by changes in clinical practice through time, population differences, and differences in how clinical information is recorded in each system [71]. ML models were relatively more adept, calibration-wise, in the presence of dataset shift. However, it is questionable whether the calibration observed is good enough to consider the models appropriate for probabilistic statements. Also, whether this improvement compensates the computational and implementation difficulties that might arise from implementing intensive ML models in practice was out of scope within in this work.

Calibration issues have consequences in how opioid risk score might need to be used in practice, as policies informed on whether patients exceed a certain threshold will be unpredictable when used in different centres, or throughout time [72], making the need for recalibration more frequent. Percentile-based policies, where individual practices identify the top percent of patients at risk of dying due to opioids, can become an alternative to threshold-based policies, monitoring or offering preventive resources in retrospect to patients which, within that particular population, are at the highest risk of experience opioid-related adverse events. Given the high recall, specificity and lower precision, the models developed here could be useful in identifying which patients in a practice have the lowest risks, which is especially informative when there are limited treatment options for pain. This can help more informed decisions about shared decision making to balance the benefits and risks of opioids for the individual. The consideration of calibration during model validation is important and has previously been raised as an issue of modern ML [73].

Whilst there are several risk prediction models being developed, very few are implemented into clinical practice. At present for non-cancer pain, it is recognised that there are limited pharmacological treatment options. Opioids are a class of drug that can be helpful in the short-term (e.g., immediately post-surgery) however is associated with serious adverse events and premature deaths in a proportion of individuals. Currently a ‘one size fits all’ approach is used without being able to incorporate a patient’s individual characteristics and contextual factors. Developing prediction models using both ML and traditional statistical techniques for opioid-related deaths, the most serious adverse outcome, in the context of rising opioid-related deaths in the UK has the potential to influence future outcomes and patient safety. The results of risk prediction models in this field could also be considered by guideline committees for risk stratification of individuals in non-cancer pain, to improve safer prescribing of these medications.

## Conclusion

This study provides valuable insights into the performance of various predictive models for survival predictions in the context of opioid use within a competing risks framework. The models demonstrated success in predicting survival outcomes, with high discrimination when identifying which patients are at higher risk. The study however also highlights the importance of designing robust validation pipelines and reporting both discriminative and calibration performance. The results show that, although ML methods perform well, their performance is not notably different from regression methods. However, the results still show how algorithms leveraging ‘big data’ could provide predictions valuable to clinicians and the healthcare system when managing the care patients which have recently started opioids. Ultimately, this research contributes to the growing body of knowledge on ML and risk prediction in the field of opioid use and offers a foundation for future studies seeking to optimize these models for practical application.

## Supporting information

S1 Text Additional methods: Drug preparation steps, sample size calculations, hyperparameter tuning. (DOCX)

S1 Table Opioid-related death ICD-10 code list. (DOCX)

S2 Table Equator checklists for clinical prediction models. (DOCX)

S3 Table Additional Results file. (DOCX)
