# Supplementary material for: Development and evaluation of machine learning algorithms for the prediction of opioid-related deaths among UK patients with non-cancer pain
Source: PLOS Digit Health. 2026 Jan 27;5(1):e0001190. doi: 10.1371/journal.pdig.0001190 (PMC12843567; doi:10.1371/journal.pdig.0001190)
Supplement: S1 Text — (DOCX) [file pdig.0001190.s001.docx]

**S1: Additional methods: Drug preparation steps, sample size calculations, hyperparameter tuning**

**Decisions made during drug preparation to derive opioid exposure and daily dose**

The following decisions were made during the execution of the drug preparation algorithm [reference 68, doi:10.1002/pds.5595]:

1. **Handle Implausible Quantities:** Set to mean for individual’s prescriptions for that drug *(option [c1] using drug_prep function in drugprepr library in R).*
2. **Handle Missing Quantities:** Set to mean for individual’s prescriptions for that drug *(option [b1] using drug_prep function in drugprepr library in R).*
3. **Handle Implausible Numerical Daily Doses:** Set to mean for individual’s prescriptions for that drug *(option [c1] using drug_prep function in drugprepr library in R).*
4. **Handle Missing Numerical Daily Doses:** Set to mean for population’s prescriptions for that drug *(option [b3] using drug_prep function in drugprepr library in R).*
5. **Clean Duration:** Set to missing if the duration exceeds 6 months. *(option [b_6] using drug_prep function in drugprepr library in R).*
6. **Select Stop Date:** Computed as the quotient of quantity and numerical daily dose (qty/ndd). *(option [c] using drug_prep function in drugprepr library in R).*
7. **Handle Stop Date:** Use mean prescription duration for that drug, for that individual *(option [b] using drug_prep function in drugprepr library in R).*
8. **Handle Multiple Prescriptions:** Replace with a prescription of duration equal to the mean *(option [b] using drug_prep function in drugprepr library in R).*
9. **Handle Overlapping Prescriptions:** Allow overlapping prescriptions (implicitly sum doses) *(option [a] using drug_prep function in drugprepr library in R).*
10. **Handle Short Gaps Between Prescriptions:** Do nothing: allow gap. *(option [a] using drug_prep function in drugprepr library in R).*

**Sample size calculation**

To calculate the maximum number of parameters allowed to use for our prediction problem if we wanted a minimum shrinkage rate of 0.9, we used the calculations of the paper by Riley et al (DOI 10.1002/sim.7992) and the R package psampsize (<https://cran.r-project.org/web/packages/pmsampsize/index.html>). The reference D-statistic was taken from the paper by Glanz et al (DOI 10.1007/s11606-017-4288-3) to be 1.56, with 121 events, 42828 patients and 10 parameters. From this, an adjusted Cox-Snell R^2^ of 0.00166. This was fed to the psampsize function, along with an event rate of (1233 patients)/(859062144 patient-days), a timepoint of interest of 730 days (2 years) and a mean follow-up of 836 days. The final EPP (number of needed events per parameter in the model) was 5.7, which, for our 1233 events, meant that we could use up to 216 parameters. Our total number of parameters under consideration is 68.

**Full list of variables used for the models**

Charlson comorbidity score; AIDS, cerebrovascular disease; chronic pulmonary disease; congestive heart disease; dementia; diabetes; diabetes with complications; hemiplegia; mild liver disease; moderate liver disease; myocardial infarction; peptic ulcer; peripheral vascular disease; renal disease; rheumatic disease; alcohol abuse; substance use disorder; depression; suicide or self-harm; prescription of benzodiazepines (in the past two years, the past 30 days, and concomitant); prescription of gabapentinoids (in the past two years and concomitant); migraines; fibromyalgia; back pain; number of GP visits; number of health utilisations; major surgery; age; opioid substance (buprenorphine, codeine, dextropropoxyphene, diamorphine, dihydrocodeine, fentanyl, morphine, oxycodone, tramadol, or other); sex; region (East Midlands, East, London, North East, North West, South Central, South East, South West, West Midlands, Yorkshire and the Humber), ethnicity (Asian, Black, Mixed, White, Other, Missing/Unknown); Townsend score (Categorical quintiles + Missing); smoking status (never, former, or current).

**Hyperparameter tuning and model development**

The hyperparameter tuning was performed in each training inner fold of the overall cross-validation without any access to the validation outer fold.

Within each set of training data, the Fine & Gray model performed 5-fold crossvalidation to find the optimal hyperparameters, with a logarithmic grid search of 15 different lambda values between the identified minimum and maximum lambda values of the LASSO regularisation. The model with the largest value of lambda such that the mean cross-validate error was within one standard error of the minimum was chosen for predictions.

The Random Forest model was also tuned with 5-fold crossvalidation, performing 15 random searches to identify the optimal hyperparameters in the following ranges:

- Number of trees: 50 – 300
- Subsample size: 16,000 – 10,000
- Variables to split per node: 5 – 15
- Minimum size of terminal node: 50 – 500
- Number of split points: 2 – 20

The choice of hyperparameters which resulted in the highest AUROC was chosen to train the final model.

The DeepHit model is tuned by split sample, using 20% of the training data to find the optimal set of hyperparameters. The choice of using split sample instead of cross-validation was informed by computational limitations, but also by the common use of this splitting rule in the machine learning field. The hyperparameter space explored was:

- Learning rate: 0.005,0.001 and 0.0001
- Class imbalance weight: 0.5 (no class imbalance weighting) and 0.99 (minority class have larger weight in the loss)
- Individual depth: 1 and 2 layers
- Shared depth: 1 and 2 layers
- Neural width: 16, 32 and 64 neurons
- Dropout rate: 0, 0.1 and 0.5

The hyperparameters were tuned sequentially to maximise the AUROC of a DeepHit network trained for 50 epochs, in the following order: Learning rate, Layer width, Shared depth, Individual depth, Class imbalance weight, DeepHit loss alpha, DeepHit loss sigma, Dropout rate, Learning rate, Layer width, Class imbalance weight. A final 5-fold cross-validation is used to identify the number of epochs for which to train the model on so that the AUROC is maximised (with a maximum of 120 epochs). The final model is trained with a batch size of 2048 for the optimal number of epochs.

The models were recalibrated to their respective inner cross-validation folds (without any access to the outer fold). This was done by calculating the average cumulative incidence function of the opioid-related death outcome and dividing it by the observed cumulative incidence function in the model’s inner-fold training data, giving a recalibration coefficient for the model at each prediction time point. The models are recalibrated at each time-point so as to match the observed incidence through time of their training data.

**Fine and Gray penalisation details**

The model was estimated using the glmnet implementation within the riskRegression framework. The penalised coefficients at the $\lambda_{1-se}$value were extracted as the final model.

Selected penalty ($\lambda_{1-se})$: ${4.72 \times10}^{-6}$

Minimum $\lambda$: ${6.56 \times10}^{-7}$

Number of $\lambda$ values tested: 15

$\lambda$ index used: 5 of 15 (i.e., the 5th regularisation step)

Cross-validation: 10-fold internal cross validation

Penalty type: LASSO (L1 regularisation)

Family: Fine-Gray (competing risks)

A complete list of non-zero coefficients for the Fine Gray model is nor provided in Supplementary Table, with corresponding variable names and penalised coefficients.

Standard errors are not directly available for penalised Fine-Gray models estimated via LASSO, as the penalty term induces variable shrinkage that invalidates classical variance estimates. We therefore report the penalised coefficients (at $\lambda_{1-se}$: ${4.72 \times10}^{-6}$) and internal validation performance.

**Fine Grey Penalisation and hyperparameters**

The LASSO penalty was selected through 10-fold cross-validation to minimise deviance. Model tuning used the default glmnet parameters, except when noted:

alpha = 1 (LASSO)

nlambda = 100

standardize = TRUE

family= “cox”

type.measure = “deviance”.

The training and validation script (R code) for model fitting and performance evaluation were uploaded to GitHub.

**Random Survival Forest description and hyperparameters**

Random Survival Forest (trained using the randomForestSRC package, model fittings and predictions were performed with rfsrc.fast() using the logrank split rule and 24 discrete time cuts (1-24 months)).

Final hyperparameters (model settings)

Number of trees: 241

Number of variables randomly selected at each split(mtry): 5

Minimum size of terminal nodes: 724

Number of random split points (nsplit): 5

**DeepHit Survival description and hyperparameters**

DeepHit survival model implemented in PyTorch Lightning, with hyperparameter tuning performed via nested 5-fold cross-validation.

Activation function: LeakyReLU.

Dropout rate: 0–0.2.

Learning rate (lr): 0.01–0.00005.

Additional DeepHit parameters: alpha = 1, sigma = 0.1
