# Supplementary material for: Development and evaluation of machine learning algorithms for the prediction of opioid-related deaths among UK patients with non-cancer pain
Source: PLOS Digit Health. 2026 Jan 27;5(1):e0001190. doi: 10.1371/journal.pdig.0001190 (PMC12843567; doi:10.1371/journal.pdig.0001190)
Supplement: S1 Table — (DOCX) [file pdig.0001190.s002.docx]

**S1 Table: Opioid-related death ICD-10 code list**

The following ICD-10 code list using linked data from the Office of National Statistics was used to identify deaths associated with opioids:

| **ICD-10** | **Description** |
| --- | --- |
| T40.0 | Poisoning by narcotics-opium |
| T40.1 | Poisoning by narcotics-heroin |
| T40.2 | Poisoning by narcotics-other (codeine, morphine) |
| T40.3 | Poisoning by narcotics-methadone |
| T40.4 | Poisoning by narcotics-pethidine |
| T40.6 | Poisoning by narcotics-other |
| T50.7 | Poisoning: Analeptics and opioid receptor antagonists |
| X42 | Accidental poisoning by and exposure to narcotics and psychodysleptics [hallucinogens], not elsewhere classified |
| T50.7 | Poisoning: Analeptics and opioid receptor antagonists |
| Y12 | Poisoning by and exposure to narcotics and psychodysleptics [hallucinogens], not elsewhere classified, undetermined intent |
| X62 | Intentional self-poisoning by and exposure to narcotics and psychodysleptics [hallucinogens], not elsewhere classified |
| F11 | Mental and behavioural disorders due to use of opioids |
| F11.0 | Mental and behavioural disorders due to use of opioids |
| F11.1 | Mental and behavioural disorders due to use of opioids |
| F11.2 | Mental and behavioural disorders due to use of opioids |
| F11.3 | Mental and behavioural disorders due to use of opioids |
| F11.4 | Mental and behavioural disorders due to use of opioids |
| F11.5 | Mental and behavioural disorders due to use of opioids |
| F11.6 | Mental and behavioural disorders due to use of opioids |
| F11.7 | Mental and behavioural disorders due to use of opioids |
| F11.8 | Mental and behavioural disorders due to use of opioids |
| F11.9 | Mental and behavioural disorders due to use of opioids |
| R78.1 | Finding of opiate drug in blood |
