# Supplementary material for: Development and evaluation of machine learning algorithms for the prediction of opioid-related deaths among UK patients with non-cancer pain
Source: PLOS Digit Health. 2026 Jan 27;5(1):e0001190. doi: 10.1371/journal.pdig.0001190 (PMC12843567; doi:10.1371/journal.pdig.0001190)
Supplement: S2 Table — (DOCX) [file pdig.0001190.s003.docx]

**S2 Table: Equator checklists for clinical prediction models**

**Table A: TRIPOD checklist for reporting of clinical prediction model development**

| **Section/Topic** | **Item** | **Checklist Item** | **Page** |
| --- | --- | --- | --- |
| **Title and abstract** | | | |
| Title | 1 | Identify the study as developing and/or validating a multivariable prediction model, the target population, and the outcome to be predicted. | 1 |
| Abstract | 2 | Provide a summary of objectives, study design, setting, participants, sample size, predictors, outcome, statistical analysis, results, and conclusions. | 1 |
| **Introduction** | | | |
| Background and objectives | 3a | Explain the medical context (including whether diagnostic or prognostic) and rationale for developing or validating the multivariable prediction model, including references to existing models. | 1-2 |
|  | 3b | Specify the objectives, including whether the study describes the development or validation of the model or both. | 2 |
| **Methods** | | | |
| Source of data | 4a | Describe the study design or source of data (e.g., randomized trial, cohort, or registry data), separately for the development and validation data sets, if applicable. | 2-3,4 |
|  | 4b | Specify the key study dates, including start of accrual; end of accrual; and, if applicable, end of follow-up. | 3,4 |
| Participants | 5a | Specify key elements of the study setting (e.g., primary care, secondary care, general population) including number and location of centres. | 2-3,4,5,6 |
|  | 5b | Describe eligibility criteria for participants. | 2-3 |
|  | 5c | Give details of treatments received, if relevant. | N/A |
| Outcome | 6a | Clearly define the outcome that is predicted by the prediction model, including how and when assessed. | 3,Supp2 |
|  | 6b | Report any actions to blind assessment of the outcome to be predicted. | 2-3 |
| Predictors | 7a | Clearly define all predictors used in developing or validating the multivariable prediction model, including how and when they were measured. | 2-3,Supp4 |
|  | 7b | Report any actions to blind assessment of predictors for the outcome and other predictors. | 2-3 |
| Sample size | 8 | Explain how the study size was arrived at. | 3,Supp3 |
| Missing data | 9 | Describe how missing data were handled (e.g., complete-case analysis, single imputation, multiple imputation) with details of any imputation method. | 3 |
| Statistical analysis methods | 10a | Describe how predictors were handled in the analyses. | 3-4 |
|  | 10b | Specify type of model, all model-building procedures (including any predictor selection), and method for internal validation. | 3 |
|  | 10d | Specify all measures used to assess model performance and, if relevant, to compare multiple models. | 2-5 |
| Risk groups | 11 | Provide details on how risk groups were created, if done. | 4 |
| **Results** | | | |
| Participants | 13a | Describe the flow of participants through the study, including the number of participants with and without the outcome and, if applicable, a summary of the follow-up time. A diagram may be helpful. | 5,6 |
|  | 13b | Describe the characteristics of the participants (basic demographics, clinical features, available predictors), including the number of participants with missing data for predictors and outcome. | Tab1 |
| Model development | 14a | Specify the number of participants and outcome events in each analysis. | Tab 1 |
|  | 14b | If done, report the unadjusted association between each candidate predictor and outcome. | Not included. |
| Model specification | 15a | Present the full prediction model to allow predictions for individuals (i.e., all regression coefficients, and model intercept or baseline survival at a given time point). | Supp 7,8 |
|  | 15b | Explain how to the use the prediction model. | 3-4 |
| Model performance | 16 | Report performance measures (with CIs) for the prediction model. | 5-6 |
| **Discussion** | | | |
| Limitations | 18 | Discuss any limitations of the study (such as nonrepresentative sample, few events per predictor, missing data). | 7-8 |
| Interpretation | 19b | Give an overall interpretation of the results, considering objectives, limitations, and results from similar studies, and other relevant evidence. | 7-9 |
| Implications | 20 | Discuss the potential clinical use of the model and implications for future research. | 9 |
| **Other information** | | | |
| Supplementary information | 21 | Provide information about the availability of supplementary resources, such as study protocol, Web calculator, and data sets. | Supp1-5 |
| Funding | 22 | Give the source of funding and the role of the funders for the present study. | 10 |

**Table B: MI-CLAIM checklist for reporting on artificial intelligence modelling**

| Description | Completed | Page Number | Notes |
| --- | --- | --- | --- |
| Study Design (Part 1) | | | |
| The clinical problem in which the model will be employed is clearly detailed in the paper. |  | 2 |  |
| The research question is clearly stated. |  | 9 |  |
| The characteristics of the cohorts (training and test sets) are detailed in the text. |  | 5 |  |
| The cohorts (training and test sets) are shown to be representative of real-world clinical settings. |  | 3 |  |
| The state-of-the-art solution used as a baseline for comparison has been identified and detailed. |  | 4 | Fine & Gray LASSO as regression baseline. |
| Data and optimization (Parts 2, 3) | | | |
| The origin of the data is described and the original format is detailed in the paper. |  | 3 |  |
| Transformations of the data before it is applied to the proposed model are described. |  | 3 |  |
| The independence between training and test sets has been proven in the paper. |  | 4,5 |  |
| Details on the models that were evaluated and the code developed to select the best model are provided. |  | 4 |  |
| Is the input data type structured or unstructured? |  | 3 |  |
| Model performance (Part 4) | | | |
| The primary metric selected to evaluate algorithm performance (e.g., AUC, F-score, etc.), including the justification for selection, has been clearly stated. |  | 4,5 |  |
| The primary metric selected to evaluate the clinical utility of the model (e.g., PPV, NNT, etc.), including the justification for selection, has been clearly stated. |  | 4,5 |  |
| The performance comparison between baseline and proposed model is presented with the appropriate statistical significance. |  | 6, 7 | Confidence intervals are used for comparisons. |
| Model examination (Part 5) | | | |
| Examination technique 1 |  | 7 | SHAP values |
| Examination technique 2 |  | 7 | Hazard ratios of regression reported instead. |
| A discussion of the relevance of the examination results with respect to model/algorithm performance is presented. |  | 8 |  |
| A discussion of the feasibility and significance of model interpretability at the case level if examination methods are uninterpretable is presented. |  | 8 | Overall examination is interpreted and discussed. |
| A discussion of the reliability and robustness of the model as the underlying data distribution shifts is included. |  | 8 |  |
| Reproducibility (Part 6): choose appropriate tier of transparency | | | |
| Tier 1: complete sharing of the code |  | 10 | Code provided to reviewers and shared on Github after publication. |
| Tier 2: allow a third party to evaluate the code for accuracy/fairness; share the results of this evaluation |  |  |  |
| Tier 3: release of a virtual machine (binary) for running the code on new data without sharing its details |  |  |  |
| Tier 4: no sharing |  |  |  |
