# Supplementary material for: Development and evaluation of machine learning algorithms for the prediction of opioid-related deaths among UK patients with non-cancer pain
Source: PLOS Digit Health. 2026 Jan 27;5(1):e0001190. doi: 10.1371/journal.pdig.0001190 (PMC12843567; doi:10.1371/journal.pdig.0001190)
Supplement: S3 Table — (DOCX) [file pdig.0001190.s004.docx]

**S3: Additional Results file**

**S3 Table 1: Coefficients of the Fine & Gray model, converted to hazard ratios**

| Chronic Pulmonary Disease | Congestive Heart Failure | Dementia | Mild Liver Disease | Moderate Liver Disease | Peripheral Vascular Disease | Rheumatological Disease | Alcohol Use | Substance Use Disorder | Suicide/Self-Harm |
| --- | --- | --- | --- | --- | --- | --- | --- | --- | --- |
| 1.537848 | 1.237269 | 0.970861 | 1.2375 | 1.105046 | 1.194332 | 1.055122 | 3.233899 | 3.213035 | 2.120843 |
| Benzodiazepine Use | Benzodiazepine Prescription (Last 30 Days) | Gabapentinoid Use (Concurrent) | Gabapentinoid Prescription (Last 2 Years) | Migraine | Back/Spinal Pain | Health Utilization | Major Surgery | Age | Dihydrocodeine |
| 1.027388 | 1.256622 | 1.007591 | 2.114937 | 0.58862 | 0.833137 | 1.01717 | 0.850277 | 1.031191 | 0.980246 |
| Morphine | Female | Never Smoked | Current Smoker | North West | South West | West Midlands | Asian | Black | Mixed Ethnicity |
| 2.450075 | 0.616155 | 0.790395 | 1.080011 | 1.059902 | 0.769948 | 1.039848 | 0.747107 | 0.812905 | 0.913738 |
| 1st Townsend Quintile (Least Deprived) | 2nd Townsend Quintile | 4th Townsend Quintile | 5th Townsend Quintile (Most Deprived) |  |  |  |  |  |  |
| 0.912288 | 0.955904 | 1.059925 | 1.139359 |  |  |  |  |  |  |

**S3 Table 2: Average difference in SHAP values for the DeepHit model of categorical variables between patients which have a positive record of the variables and those which do not, sorted from largest to lowest**

| Fibromyalgia | 0.018154 | Other Ethnicity | 0.000129 |
| --- | --- | --- | --- |
| Oxycodone | 0.011804 | 4th Townsend Quintile | 0.000103 |
| Gabapentinoid Prescription (Last 2 Years) | 0.010073 | North West | 8.05E-05 |
| Buprenorphine | 0.008168 | Charlson Score | 6.91E-05 |
| Dementia | 0.007967 | Black | 6.49E-05 |
| Diamorphine | 0.00471 | South East Coast | 5.36E-05 |
| Fentanyl | 0.00438 | NA | 2.96E-05 |
| Morphine | 0.002861 | West Midlands | 2.91E-05 |
| Alcohol Use | 0.001551 | North East | 1.93E-05 |
| Moderate Liver Disease | 0.001349 | Migraine | 1.51E-05 |
| Age | 0.00126 | White | 2.16E-06 |
| Dextropropoxyphene | 0.001238 | South West | -1.33E-05 |
| Congestive Heart Failure | 0.001029 | Diabetes with Complications | -1.37E-05 |
| Hemiplegia | 0.001002 | Never Smoked | -1.72E-05 |
| Other Opioid | 0.000859 | Back/Spinal Pain | -1.85E-05 |
| Substance Use Disorder | 0.000855 | South Central | -1.86E-05 |
| Mild Liver Disease | 0.000762 | 3rd Townsend Quintile | -2.10E-05 |
| Tramadol | 0.00076 | Female | -3.58E-05 |
| Dihydrocodeine | 0.000753 | Asian | -3.78E-05 |
| Rheumatological Disease | 0.000613 | Mixed Ethnicity | -6.04E-05 |
| Total GP Visits | 0.000501 | East of England | -6.52E-05 |
| Benzodiazepine Prescription (Last 30 Days) | 0.000492 | Health Utilization | -7.58E-05 |
| Peripheral Vascular Disease | 0.000491 | Renal Disease | -1.00E-04 |
| Codeine | 0.000403 | Former Smoker | -0.00011 |
| 5th Townsend Quintile (Most Deprived) | 0.000399 | AIDS | -0.00012 |
| Peptic Ulcer | 0.000376 | 2nd Townsend Quintile | -0.00012 |
| Gabapentinoid Use (Concurrent) | 0.000294 | London | -0.00021 |
| Cerebrovascular Disease | 0.000287 | Major Surgery | -0.00023 |
| Myocardial Infarction | 0.000275 | 1st Townsend Quintile (Least Deprived) | -0.00023 |
| Benzodiazepine Use | 0.000258 | Yorkshire and the Humber | -0.00024 |
| Suicide/Self-Harm | 0.000253 | Ethnicity Missing | -0.00043 |
| Current Smoker | 0.000177 | Townsend Quintile Missing | -0.00047 |
| Depression | 0.000164 | East Midlands | -0.0005 |
| Diabetes | 0.000145 | Benzodiazepine Prescription (Last 2 Years) | -0.00059 |
| Chronic Pulmonary Disease | 0.000134 |  |  |

**S3 Figure 1: SHAP plot of the five continuous numerical variables used in the DeepHit model**


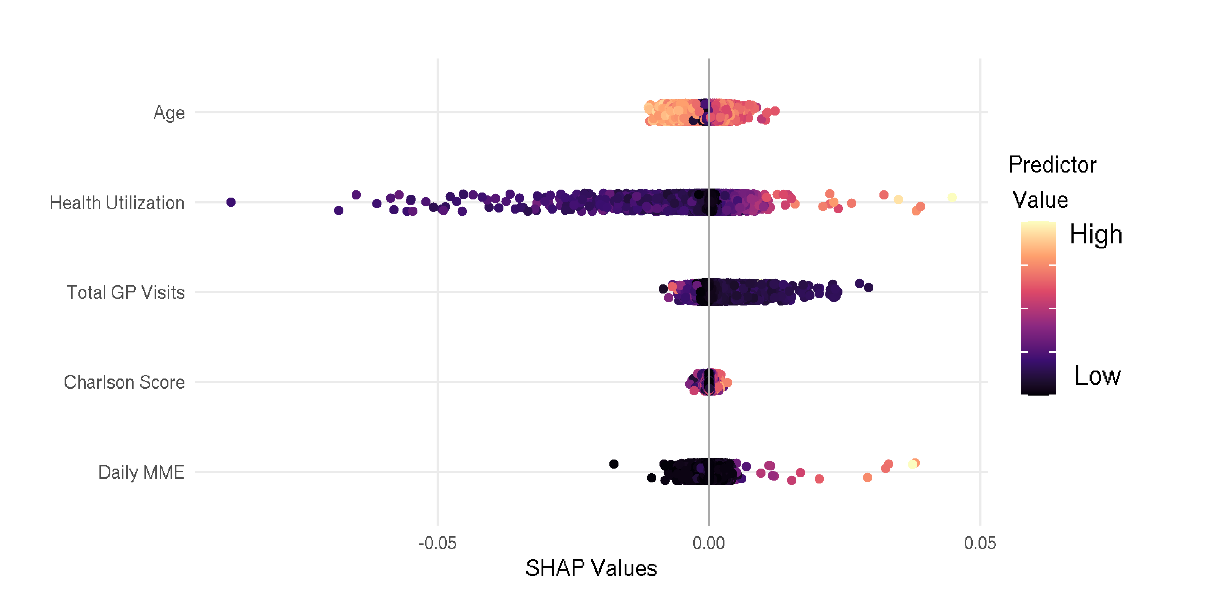


**S3 Table 3: Example of ten predictions given by each model, chosen from each decile of the risk score predictions given by the DeepHit model. The last column includes SHAP interpretability values from the DeepHit model**

| Decile of Risk Score | Predictions | | | SHAP (DeepHit) |
| --- | --- | --- | --- | --- |
|  | Fine & Gray | Random Forest | DeepHit |  |
| 1st | 0.000164 | 6.07E-05 | 0.000513 | **Prediction increased by:** Age = 29.00 -> 0.00191 Region (Not From North West) -> 0.00011 Charlson Score = 0 -> 0.00009  **Prediction decreased by:** Townsend Quintile = 1 -> -0.00016 No Renal Disease -> -0.00014 |
| 2nd | 0.000484 | 0.000325 | 0.000643 | **Prediction increased by:** No Benzodiazepines in Last 30 Days -> 0.00045 Not from Townsend Quintile = 5 -> 0.00030 No COPD -> 0.00010 Charlson Score = 0 -> +0.00007  **Prediction decreased by:** Age = 62 -> -0.00096 Townsend Not Missing -> -0.00036 |
| 3rd | 0.000204 | 0.000183 | 0.000724 | **Prediction increased by:** Age = 45.00 -> 0.00078  **Prediction decreased by:** Not from 1^st^ Townsend Quintile -> -0.00021 Daily MME = 10.670176 -> -0.00017 |
| 4th | 0.000801 | 0.000535 | 0.000736 | **Prediction increased by:** No Dihydrocodeine at Rx -> 0.00085 Age = 55.00 -> 0.00081 Not Currently Smoking -> 0.00027 No Depression -> 0.00013 Never Smoker -> 0.00009  **Prediction decreased by:** Codeine at Rx -> -0.00048 Daily MME = 19.940979 -> -0.00031 |
| 5th | 0.000651 | 0.000487 | 0.000854 | **Prediction increased by:** Not Townsend Quintile = 5 -> 0.00050  **Prediction decreased by:** Ethnicity Not Missing -> -0.00060 Total GP Visits = 8.000000 -> -0.00016 |
| 6th | 0.001149 | 0.00047 | 0.000872 | **Prediction decreased by:** Age = 63.000000 -> -0.00047 |
| 7th | 0.000809 | 0.000503 | 0.000955 | **Prediction increased by:** Total GP Visits = 3.00 -> 0.00041 Not Townsend Quintile = 5 -> 0.00040 Age = 64.00 -> 0.00037  **Prediction decreased by:** Not from East of England -> -0.00019 Daily MME = 27.418280 -> -0.00014 Health Utilisations = 18.000000 -> -0.00012 |
| 8th | 0.001053 | 0.001666 | 0.001044 | **Prediction increased by:** No Tramadol at Incidence -> 0.00107 No Benzodiazepine in Last 30 Days -> 0.00085 Age = 65.00 -> 0.00055 Health Utilisations = 34.00 -> 0.00043 From North West = 1.00 -> 0.00027  **Prediction decreased by:** No Benzodiazepine in Last 2 Year -> -0.00073 No Codeine at Incidence -> -0.00047 Total GP Visits = 19.000000 -> -0.00034 Not from 1^st^ Townsend Quintile -> -0.00033 |
| 9th | 0.002596 | 0.002136 | 0.001255 | **Prediction increased by:** Health Utilisations = 28.00 -> 0.00038 No Depression -> 0.00022 Not from 4^th^ Townsend Quintile -> 0.00009  **Prediction decreased by:** Total GP Visits = 12.000000 -> -0.00054 Not from East of England -> -0.00016 |
| 10th | 0.002074 | 0.003282 | 0.001416 | **Prediction increased by:** Age - 62 -> 0.00057 No Concurrent Gabapentinoid Use -> 0.00050 Not from South East Coast -> 0.00010 Female -> 0.00007 No Depression -> 0.00005  **Prediction decreased by:** Total GP Visits = 7.000000 -> -0.00019 2^nd^ Townsend Quintile -> -0.00016 |

**S3 Table 4: Performance metrics after recalibration to the AURUM external cohort (intercept-only)**

*Overall performance across 4-24 months*

| **Model** | **Mean AUROC (%)** | **Mean AUPRC (%)** |
| --- | --- | --- |
| Random Survival Forest | 81.3% | 0.35% |
| DeepHit | 81.1% | 0.32% |

*AUROC Performance at specific horizons (6,12,24 months)*

| **Model** | **6 months** | **12 months** | **24 months** |
| --- | --- | --- | --- |
| RSF | **0.837** | **0.816** | **0.796** |
| DeepHit | **0.835** | **0.808** | **0.789** |

*Calibration – Expected/Observed (E/O) ratios after recalibration*

| **Model** | **6 months** | **12 months** | **24 months** |
| --- | --- | --- | --- |
| RSF | **0.99** | **0.94** | **1.00** |
| DeepHit | **1.03** | **1.01** | **1.01** |

*Calibration slopes*

| **Model** | **6 months** | **12 months** | **24 months** |
| --- | --- | --- | --- |
| RSF | **1.13** | **1.01** | **0.91** |
| DeepHit | **1.09** | **0.90** | **0.57** |

**S3 Table 5: Performance metrics after recalibration to clinically relevant subgroups (under 60 years-old in the external cohort) (intercept-only)**

*Overall performance across 4-24 months*

| **Model** | **Mean AUROC (%)** | **Mean AUPRC (%)** |
| --- | --- | --- |
| Fine–Gray | 77.8 (CI wide: 61.5–94.0) | 0.58(baseline 0.04) |
| Random Survival Forest | 76.9 (69.2–84.7) | 0.28 (0.14–0.42) |
| DeepHit | 79.7 (70.7–88.6) | 0.25(approx. 0–0.54) |

*AUROC Performance at specific horizons (6,12,24 months)*

| **Model** | **6 months** | **12 months** | **24 months** |
| --- | --- | --- | --- |
| FG | **0.85 (0.68–1.02)** | **0.80 (0.65–0.96)** | **0.70 (0.59–0.82)** |
| RSF | **0.84 (0.71–0.96)** | **0.75 (0.67–0.83)** | **0.77 (0.66–0.89)** |
| DeepHit | **0.83 (0.76–0.91)** | **0.80 (0.70–0.90)** | **0.76 (0.68–0.84)** |

*Calibration – Expected/Observed (E/O) ratios after recalibration*

| **Model** | **6 months** | **12 months** | **24 months** |
| --- | --- | --- | --- |
| FG | **0.10** | **0.11** | **0.14** |
| RSF | **0.18** | **0.18** | **0.16** |
| DeepHit | **0.18** | **0.19** | **0.17** |

*Calibration slopes*

| **Model** | **6 months** | **12 months** | **24 months** |
| --- | --- | --- | --- |
| FG | **0.36** | **0.36** | **0.36** |
| RSF | **1.15** | **0.97** | **1.07** |
| DeepHit | **1.34** | **1.10** | **0.58** |

**S3 Table 6: Performance metrics after recalibration to clinically relevant subgroups (60 years-old and older, in the external cohort) (intercept-only)**

*Overall performance across 4-24 months*

| **Model** | **Mean AUROC (%)** | **Mean AUPRC (%)** |
| --- | --- | --- |
| **Fine–Gray** | **79.4** (75.4–83.5) | **0.58** (0.43–0.73) |
| **Random Survival Forest** | **79.3** (74.2–84.3) | **0.47** (0.33–0.60) |
| **DeepHit** | **77.2** (71.6–82.9) | **0.44** (0.30–0.58) |

*AUROC Performance at specific horizons (6,12,24 months)*

| **Model** | **6 months** | **12 months** | **24 months** |
| --- | --- | --- | --- |
| **Fine–Gray** | 0.79 (0.73–0.85) | 0.80 (0.76–0.84) | 0.78 (0.73–0.83) |
| **RSF** | 0.81 (0.77–0.85) | 0.79 (0.75–0.84) | 0.78 (0.71–0.84) |
| **DeepHit** | 0.79 (0.69–0.89) | 0.77 (0.68–0.85) | 0.74 (0.68–0.81) |

*Calibration – Expected/Observed (E/O) ratios after recalibration*

| **Model** | **6 months** | **12 months** | **24 months** |
| --- | --- | --- | --- |
| **Fine–Gray** | 0.14 | 0.19 | 0.26 |
| **RSF** | 0.34 | 0.39 | 0.46 |
| **DeepHit** | 0.44 | 0.56 | 0.66 |

*Calibration slopes*

| Model | 6 months | 12 months | 24 months |
| --- | --- | --- | --- |
| **Fine–Gray** | 0.28 | 0.29 | 0.31 |
| **RSF** | 1.02 | 0.93 | 0.91 |
| **DeepHit** | 1.05 | 0.88 | 0.55 |
